# Supplementary material for: Interaction between β-lactoglobulin and EGCG under high-pressure by molecular dynamics simulation
Source: PLoS One. 2021 Dec 21;16(12):e0255866. doi: 10.1371/journal.pone.0255866 (PMC8691620; doi:10.1371/journal.pone.0255866)
Supplement: S4 File — (DOCX) [file pone.0255866.s004.docx]

**Fig 7. The 2-D plot for interaction between EGCG and β-lactoglobulin at site** 1 **under 0.1 MPa (a) and 600 MPa (b)**


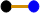
 Non-ligand bond
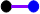
 Ligand bond
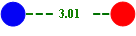
 Hydrogen bond and its length


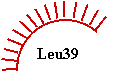
Non-ligand residues involved in hydrophobic


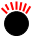
Corresponding atoms involved in hydrophobic contact(s)

**Fig 8. The 2-D plot for interaction between EGCG and β-lactoglobulin at site** 2 **under 0.1 MPa (a) and 600 MPa (b)**


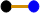
 Non-ligand bond
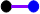
 Ligand bond
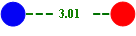
 Hydrogen bond and its length


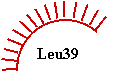
Non-ligand residues involved in hydrophobic


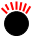
Corresponding atoms involved in hydrophobic contact(s)
