# Supplementary material for: Interaction between β-lactoglobulin and EGCG under high-pressure by molecular dynamics simulation
Source: PLoS One. 2021 Dec 21;16(12):e0255866. doi: 10.1371/journal.pone.0255866 (PMC8691620; doi:10.1371/journal.pone.0255866)
Supplement: S1 Data — (DOCX) [file pone.0255866.s005.docx]

**Highlights**

● β-lactoglobulin and EGCG binding under 600 MPa by molecular dynamics simulation.

● 600 MPa result in reduction of binding energy between EGCG and β-lactoglobulin.

● The best binding site of EGCG was in the hydrophobic cavity at atmospheric pressure.

● Binding free energy in protein surface was higher than hydrophobic cavity at 600 MPa.

● Binding site of EGCG on the protein surface shifted significantly at 600 MPa.
