# Supplementary figures and images for: Interaction between β-lactoglobulin and EGCG under high-pressure by molecular dynamics simulation
Source: PLoS One. 2021 Dec 21;16(12):e0255866. doi: 10.1371/journal.pone.0255866 (PMC8691620; doi:10.1371/journal.pone.0255866)

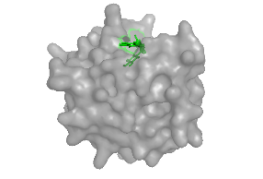

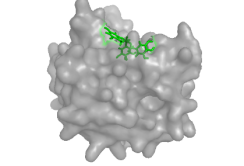


-109.411 kJ/mol

-75.858 kJ/mol

600MPa


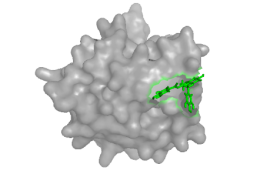

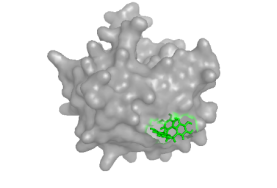


-99.148 kJ/mol

-81.107 kJ/mol

600MPa


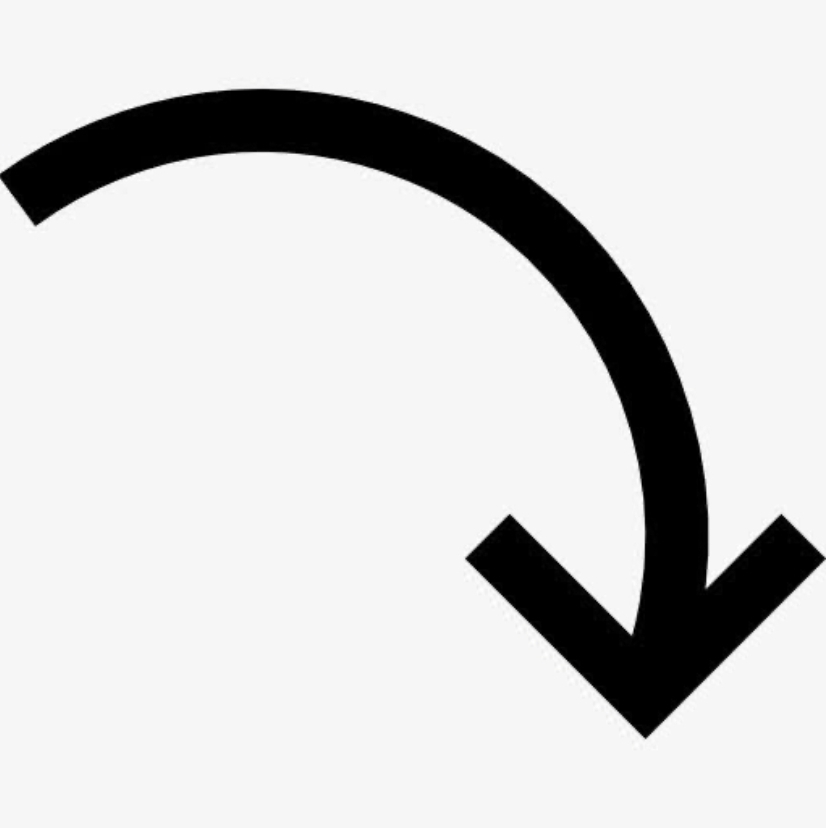

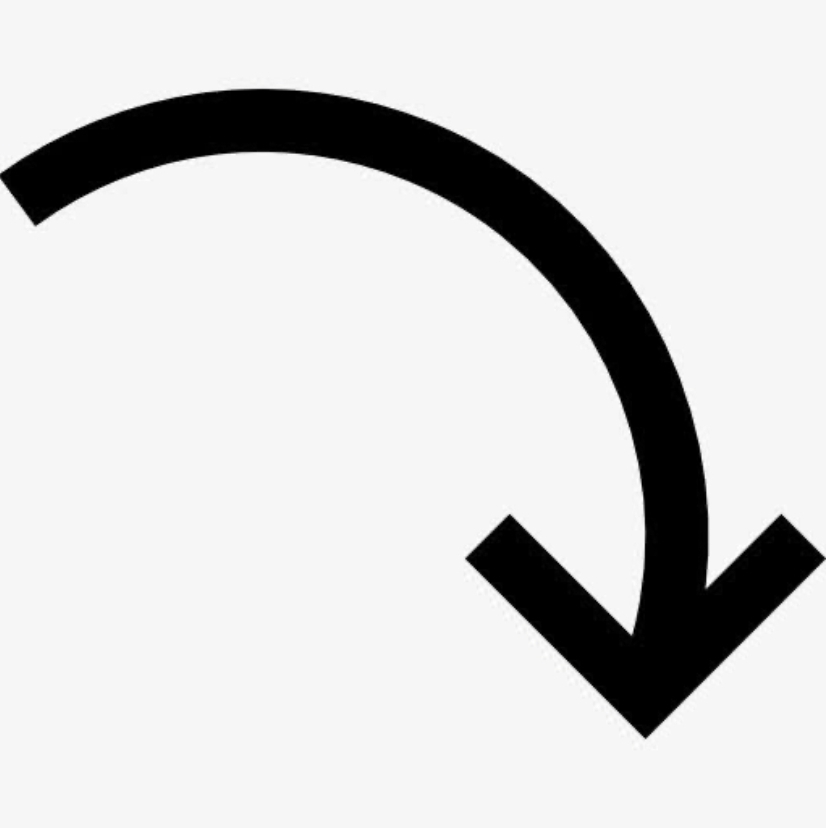


Site1


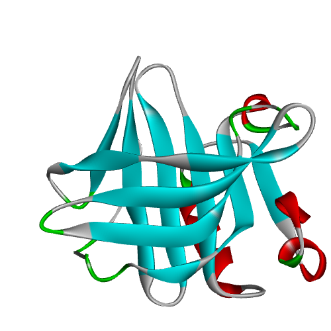

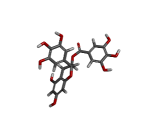


Site2

Supplement: S1 Graphical abstract — (DOCX) [file pone.0255866.s006.docx]
